# Supplementary material for: A Combined Administration of Testosterone and Arginine Vasopressin Affects Aggressive Behavior in Males
Source: Brain Sci. 2021 Dec 9;11(12):1623. doi: 10.3390/brainsci11121623 (PMC8699569; doi:10.3390/brainsci11121623)
Supplement: Supplementary file 1 [file brainsci-11-01623-s001.zip › brainsci-1409182-supplementary.pdf]

Supplementary material

To test if the effects of the T/AVP administration were different from the administration of the same dose of testosterone (T) as applied in the combined study branch, we compared the data against a group of healthy males selected from a previous study with a similar design. Since the comparison sample included 54 participants that received T administration, we selected 19 participants of who met the criteria to have indicated to receive T or PL (belief) and who were not excluded from the fMRI analyses due to extensive movement. The remaining participants were than matched by age to the treatment group of the T/AVP data set using propensity score matching. Mean age did not differ between all three groups (T/AVP, PL and T) ($F(2,56)= .331$, $p =.720$). A neuropsychological test measuring verbal intelligence with German vocabulary (Wortschatztest (WST)) also did not differ between all three groups ($F(2,56)= .237$, $p =.790$). The standard error that was used for propensity matching was 0.07. All steps of analyses that included the preprocessing of the data and operationalization of the variables were kept parallel to the T/AVP data set reported in the manuscript.

Hormonal data analysis

The results for the hormonal analysis including three administration groups (T/AVP, PL, T) and three times points (T1 = pre administration, T2= 3.5 h after administration/ pre task, T3 = after the task) are presented in Table S1. Bonferroni corrected post hoc comparisons are presented in Table S2.

Table S1. Statistical effects of the hormonal analysis according to the 3 group model

	F	p	Np^2
Time	**11.08***	**.002**	**.25**
Administration x Time	**5.98***	**.006**	**.28**
Belief x Time	0.19	.594	.01
Administration x Belief x Time	0.10	.908	.01
Administration	**6.15***	**.005**	**.90**
Belief	1.25	.271	.04
Administration x Belief	0.63	.538	.04

Note: Significant effects are indicated in bold letters. *p <.05

Table S2. Post hoc tests for the interaction of time x administration in the 3-group model

Factor	contrast	M (diff)	SE	p	CI [LB-UB]	
	T1 vs T2	**-3.06***	**1.14**	**.033**	**-5.92**	**-0.20**
	T1 vs T3	**-4.84***	**1.45**	**.006**	**-8.49**	**-1.18**
	T2 vs T3	-1.77	0.79	.091	-3.75	0,20
T1	T vs PL	2.18	2.76	1.00	-4.77	9.13
	T vs T/AVP	3.38	2.82	.718	-3.73	10.48
	PL vs T/AVP	1.20	2.54	1.00	-5.19	7.59
T2	T vs PL	**10.85***	**2.81**	**.001**	**3.79**	**17.92**
	T vs T/AVP	**7.47***	**2.87**	**.041**	**0.25**	**14.69**
	PL vs T/AVP	-3.38	2.58	.595	-9.88	3.11
T3	T vs PL	**13.36***	**3.43**	**.001**	**4.73**	**21.99**
	T vs T/AVP	5.38	3.50	.402	-3.44	14.19
	PL vs T/AVP	**-7.98***	**3.15**	**.048**	**-15.91**	**-0.05**
T	1 vs 2	**-7.32***	**2.20**	**.006**	**-12.85**	**-1.78**
	1 vs 3	**-9.23***	**2.81**	**.007**	**-16.30**	**-2.15**
	2 vs 3	-1.91	1.52	.649	-5.73	1.91
PL	1 vs 2	1.36	1.80	1.00	-3.16	5,88
	1 vs 3	1.95	2.29	1.00	-3.82	7.72

	2 vs 3	0.59	1.24	1.00	-2.52	3.71
T/AVP	1 vs 2	-3.23	1.89	.292	-7.99	1.54
	1 vs 3	**-7.23***	**2.42**	**.015**	**-13.32**	**-1.14**
	2 vs 3	**-4.00***	**1.31**	**.013**	**-7.29**	**-0.72**

Note: Significant effects are indicated in bold letters. *p <.05 (Bonferroni corrected)

Behavioral analysis

The results for the analysis of money subtractions including the between subject factor administration groups (group1 = T/AVP, group 2 = PL, group 3 = T), belief about the administration (hormone, no hormone) and two conditions (provocation = loss, no provocation = win) are presented in Table S3.

Table S3. Statistical effects for the TAP behavioral analysis according to the 3-group model (T/AVP, PL, T)

Effects	F	p	ηp^2
Provocation	2.50	.120	0.045
Provocation * administration group	2.72	.075	0.093
Provocation * belief	1.68	.201	0.031
Provocation * administration group * belief	3.05	.056	0.103
Administration group	0.02	.984	0.001
Belief	**7.40**	**.009***	**0.122**
administration group * belief	0.15	.863	0.006

Note: Significant effects are indicated in bold letters. * p <.05

Similar to the 2-group model the belief to have received any hormones was associated with higher monetary subtractions (M = 63.91 +/- 8.96) than the belief to have received placebo (M=36.94 +/- 4.25).

fMRI group analysis

The fMRI analysis for the 3 group model was carried out parallel to the two full factorial models comparing T/AVP and PL, but included a third group (testosterone administration, T). Since the analysis specifically aimed to characterize the treatment effect, which was found in the feedback period, the main effect of treatment was tested using a small volume correction by applying a mask of the effect in the 2-group model.

The main effect of the 3-group model in the feedback period and the decision period was similar to the main effect in the 2-group model (see Tables S4 and S5). There were no significant effects regarding treatment or the interaction of condition and treatment on a whole brain level if applying FWE correction. Applying small volume correction with an inclusive mask of the clusters that reflected the main effect of treatment in the 2-group model, a significantly increased activation of the T/AVP group in contrast to the T group was identified.

Table S4. MNI coordinates (x, y, z) of the peak voxel for each cluster with the size k that showed significantly increased activation during the feedback period of the win> loss and loss > win contrast across all three groups (T/AVP, PL, T)

Contrast	Region	k	T	x	y	z
Win> loss	L Precentral Gyrus	14357	14.23	-38	-12	50
	R Posterior-Medial Frontal		13.99	2	0	56

	L Posterior-Medial Frontal		13.92	-6	-8	64
	L Paracentral Lobule		11.36	-10	-26	50
	R Precentral Gyrus		11.26	42	-4	50
	RPrecentral Gyrus		11.08	38	-10	50
	R MCC		10.77	6	8	44
	L Insula Lobe	767	8.05	-34	22	6
	L Rolandic Operculum		7.84	-50	4	4
	R IFG (p. Opercularis)	728	8.59	38	14	10
	R Insula Lobe		8.37	34	24	8
	R Rolandic Operculum		6.46	50	2	4
	R Middle Temporal Gyrus	354	9.64	42	-68	8
	R Superior Temporal Gyrus	286	6.37	66	-38	14
	R SupraMarginal Gyrus		5.78	54	-40	26
	R Putamen	220	8.02	18	12	-6
	R Caudate Nucleus		7.82	12	12	-10
	L Putamen	198	8.19	-20	12	-8
	L Rolandic Operculum	151	7.39	-40	-30	22
	L Middle Occipital Gyrus	116	6.35	-46	-80	20
	L Middle Temporal Gyrus		6.18	-44	-68	12
	L Inferior Occipital Gyrus	18519	21.15	-34	-90	-10
	R Inferior Occipital Gyrus		20.77	28	-98	-6
	R Lingual Gyrus		19.74	22	-100	-12
	L Calcarine Gyrus		10.72	2	-72	16
	R Inferior Temporal Gyrus		9.57	52	-62	-18
	L Medial Temporal Pole	1391	7.33	-40	22	-34
	L Temporal Pole		6.85	-42	22	-24
	L Precentral Gyrus		6.66	-46	12	30
	L IFG (p. Triangularis)		6.04	-56	22	18
	L Inferior Temporal Gyrus		6.00	-52	-2	-36
	L Middle Temporal Gyrus		5.81	-56	4	-34
	L Middle Frontal Gyrus		5.56	-46	20	38
	L Inferior Parietal Lobule	1244	8.07	-34	-68	48
Loss> win	L Angular Gyrus		6.20	-40	-54	34
	R Superior Parietal Lobule	1125	8.08	34	-72	48
	R Angular Gyrus		7.13	44	-62	26
	R Middle Occipital Gyrus		6.99	38	-64	34
	R Inferior Temporal Gyrus	828	8.32	50	-4	-36
	R Medial Temporal Pole		7.53	48	16	-26
	R IFG (p. Orbitalis)		6.59	48	22	-14
	R Temporal Pole		5.75	36	20	-32
	R Fusiform Gyrus		5.58	36	-4	-34
	R Temporal Pole		5.24	32	18	-30
	R Precuneus	414	9.03	2	-56	74
	R Superior Medial Gyrus	321	6.92	12	52	42
	R Superior Orbital Gyrus	263	7.89	12	62	-18
	R Middle Orbital Gyrus		5.81	30	60	-12

L Middle Orbital Gyrus	234	6.09	-34	60	-12
L Superior Medial Gyrus		5.09	-4	68	0
R Middle Frontal Gyrus	160	6.47	38	20	56
L Inferior Parietal Lobule	152	7.97	-52	-40	58
L Posterior-Medial Frontal	115	7.26	-6	22	64
R Superior Parietal Lobule	98	6.22	48	-40	60
R Inferior Parietal Lobule		6.22	52	-32	56
R Postcentral Gyrus		5.20	42	-42	64
L Superior Frontal Gyrus	37	5.15	-22	58	22
R Superior Medial Gyrus	36	5.88	6	30	62
R IFG (p. Triangularis)	12	5.50	52	32	30
R IFG (p. Orbitalis)	10	5.49	42	38	-18
L Superior Medial Gyrus	7	5.01	-8	64	24

Note: L = left, R = right

Table S5. MNI coordinates (x, y, z) of the peak voxel for each cluster with the size k that showed significantly increased activation during the decision period of the win> loss and loss > win contrast across all three groups (T/AVP, PL, T)

Contrast	Region	k	T	x	y	z
Win > loss	R Middle Temporal Gyrus	46997	11.73	40	-70	10
	R Caudate Nucleus		11.20	8	12	-10
	R Middle Occipital Gyrus		10.83	44	-78	12
	L Precentral Gyrus		10.78	-38	-12	48
	L Middle Temporal Gyrus		10.69	-44	-68	10
	L Putamen		10.66	-18	12	-8
	R Cuneus		10.31	18	-78	40
	L Insula Lobe		10.28	-40	0	8
	L Middle Temporal Gyrus		10.24	-50	-66	6
	L Rolandic Operculum		10.20	-54	4	6
	L SupraMarginal Gyrus	1233	7.88	-66	-30	28
	L Superior Temporal Gyrus		7.23	-58	-30	20
	L Postcentral Gyrus		6.84	-64	-18	20
	L Thalamus	85	5.99	-6	-24	8
	L Thalamus		5.08	-8	-18	2
	R Cerebelum (Crus 1)	65	6.28	52	-58	-36
	R Cerebelum (Crus 1)		6.04	48	-56	-36
	R Cerebelum (VI)	47	5.66	26	-52	-24
	L Cerebelum (Crus 1)	41	6.23	-52	-56	-40
	L Cerebelum (Crus 1)		5.33	-38	-52	-36
	R Thalamus	38	5.64	8	-18	2
Loss > win	L Inferior Occipital Gyrus	18519	21.15	-34	-90	-10
	R Inferior Occipital Gyrus		20.77	28	-98	-6
	R Lingual Gyrus		19.74	22	-100	-12
	L Calcarine Gyrus		10.72	2	-72	16
	L Medial Temporal Pole	1391	7.33	-40	22	-34
	L Temporal Pole		6.85	-42	22	-24

L Precentral Gyrus		6.66	-46	12	30
L IFG (p. Triangularis)		6.04	-56	22	18
L Inferior Temporal Gyrus		6.00	-52	-2	-36
L Middle Temporal Gyrus		5.81	-56	4	-34
L Middle Frontal Gyrus		5.56	-46	20	38
L Inferior Parietal Lobule	1244	8.07	-34	-68	48
L Angular Gyrus		6.20	-40	-54	34
R Superior Parietal Lobule	1125	8.08	34	-72	48
R Angular Gyrus		7.13	44	-62	26
R Middle Occipital Gyrus		6.99	38	-64	34
R Inferior Temporal Gyrus	828	8.32	50	-4	-36
R Medial Temporal Pole		7.53	48	16	-26
R IFG (p. Orbitalis)		6.59	48	22	-14
R Temporal Pole		5.75	36	20	-32
R Fusiform Gyrus		5.58	36	-4	-34
R Precuneus	414	9.03	2	-56	74
R Superior Medial Gyrus	321	6.92	12	52	42
R Superior Orbital Gyrus	263	7.89	12	62	-18
R Middle Orbital Gyrus		5.81	30	60	-12
L Middle Orbital Gyrus	234	6.09	-34	60	-12
L Superior Medial Gyrus		5.09	-4	68	0
R Middle Frontal Gyrus	160	6.47	38	20	56
L Inferior Parietal Lobule	152	7.97	-52	-40	58
L Posterior-Medial Frontal	115	7.26	-6	22	64
R Superior Parietal Lobule	98	6.22	48	-40	60
R Inferior Parietal Lobule		6.22	52	-32	56
R Postcentral Gyrus		5.20	42	-42	64
L Superior Frontal Gyrus	37	5.15	-22	58	22
R Superior Medial Gyrus	36	5.88	6	30	62
R Superior Frontal Gyrus		4.97	16	34	56
R IFG (p. Triangularis)	12	5.50	52	32	30
R IFG (p. Orbitalis)	10	5.49	42	38	-18
L Superior Medial Gyrus	7	5.01	-8	64	24

Note: L = left, R = right

Significant peak voxel for the specific T/AVP > T contrast at a small volume corrected cluster level threshold in the feedback period were located at x=-52, y= -40, z=58 ($t(108)=4.00$, p= .009) and at x=-52, y= 6, z=28 ($t(108)=3.88$, p= .031). The contrast T > T/AVP did not reveal any significant activation.
